# The protracted neurotoxic consequences in mice of developmental exposures to inhaled iron nanoparticles alone or in combination with SO_2_

**DOI:** 10.3389/fnbeh.2025.1544974

**Published:** 2025-07-25

**Authors:** Deborah A. Cory-Slechta, Elena Marvin, Kevin Welle, Gunter Oberdörster, Marissa Sobolewski

**Affiliations:** Department of Environmental Medicine, University of Rochester Medical Center, Rochester, NY, United States

**Keywords:** iron, behavioral experience, brain, sulfur dioxide, glutamate, dopamine, serotonin, trans-sulfuration

## Abstract

**Introduction:**

Air pollution (AP) has been associated with increased risk for multiple neurodevelopmental disorders. As one of the most abundant contaminants of AP, iron (Fe) is critical to brain function, with both deficiencies and excesses leading to potential neurotoxicity. Our prior studies examining the impact of developmental exposures of mice to inhaled Fe (1.0 μg/m^3^) alone or in conjunction with sulfur dioxide SO_2_ (1.31 mg/m^3^; FeS) from postnatal days (PND) 4–7 and 10–13 (human 3rd trimester brain equivalent period) revealed alterations in brain neurotransmitter levels at PND14 which had generally recovered by PND60, but which were, nevertheless, followed by behavioral impairments. The current study sought to determine whether subsequent behavioral experience, which requires neurochemical mediation, had unmasked residual deficits in neurotransmitter function in response to developmental FeS or Fe inhalation.

**Methods:**

Consequently, levels of brain neurotransmitters and trans-sulfuration markers were measured in mice that had either behavioral experience (BE) or no behavioral experience (NB) at PND 215 (Fe only) or 357 (FeS).

**Results:**

BE itself markedly increased brain neurotransmitter and trans-sulfuration marker levels, particularly in males. These increases were prevented in males in both frontal cortex and striatum by prior developmental FeS exposures. In females, developmental Fe exposure was associated with residual increases particularly in striatal serotonergic function and levels of homocysteine independently of behavioral experience.

**Discussion:**

Collectively, these findings show the ability of behavioral experience to unmask later life residual consequences of developmental exposures to FeS in males and of latent emerging effects of Fe in females. The collective findings may have relevance to later life neurodegenerative diseases and disorders now increasingly associated with air pollution exposures, and also underscore how understanding how various components of air pollution influence brain is critical to regulatory decisions for public health protection.

## Introduction

A substantive literature has now accumulated that links early air pollution exposure with altered neurodevelopment. Such alterations have included structural and functional variations in brain as reported in studies from the U.S., the Netherlands, Spain and the United Kingdom (Morrel et al., 2024). Functional consequences have included such effects as increased risk of impaired motor functions (Chen et al., 2024; Whitworth et al., 2024; Shih et al., 2023; Xu et al., 2022) and general cognitive functions (Hsu et al., 2024; Chiu et al., 2023; Morgan et al., 2023), some of which were male-biased (Guilbert et al., 2023). Additionally, air pollution exposures have been found to increase risk for neurodevelopmental disorders, including autism spectrum disorder (Murphy et al., 2024; O’Sharkey et al., 2024; Carter et al., 2023; Carter et al., 2022; Dutheil et al., 2021; Yu et al., 2023; Ghahari et al., 2023), attention deficit hyperactivity disorder (Shim et al., 2022; Chang et al., 2022; Thygesen et al., 2020; Zhao et al., 2024; Fan et al., 2022) and schizophrenia (Song et al., 2023; Antonsen et al., 2020; Nobile et al., 2023; Liu et al., 2024; Zhang et al., 2024), all of which are male-biased disorders.

In correspondence with such reports, our studies in mice of developmental exposures to concentrated inhaled ambient air pollution, specifically the ultrafine particulate matter (UFP) component of air pollution, have revealed various features of neurodevelopmental disorders (Cory-Slechta et al., 2020). Among such features following early postnatal exposures (equivalent to human third trimester brain development; Clancy et al., 2007) have been ventriculomegaly, elevated brain glutamate levels, reductions in size and demyelination of corpus callosum, increases in inflammatory cytokine levels, and behavioral deficits including impulsive-like behavior, effects that were also male-biased (Allen et al., 2013; Allen et al., 2014a; Allen et al., 2014b; Allen et al., 2015) and seen in neurodevelopmental disorders (Cory-Slechta et al., 2020).

Another feature observed following UFP exposures in mice was brain metal dyshomeostasis (Cory-Slechta et al., 2020; Cory-Slechta et al., 2019), consistent with the presence of trace elements and metals in air pollution exposures (Reff et al., 2009). Brain metal dyshomeostasis has been implicated in numerous neurological and neurodevelopmental conditions (Garza-Lombó et al., 2018). Among these metals, iron (Fe) tends to be present in great abundance in the atmosphere, and was also found in excess in brains in our studies following exposures of mice to inhaled UFPs (Cory-Slechta et al., 2019). Although Fe is critical to brain development and function, excess Fe can have adverse consequences (D’Mello and Kindy, 2020; David et al., 2023), and elevated brain Fe levels are seen in multiple neurodegenerative diseases and disorders (David et al., 2023). Fe is redox-active, and elevated levels of Fe can lead to ferroptosis, an Fe-dependent form of cell death characterized by lipid peroxidation and increased intracellular Fe (Costa et al., 2023). The extent to which air pollution exposure effects may reflect persistent consequences of early life Fe inhalation via air pollution or lifelong exposures has yet to be considered.

To address such questions, we examined the impact of early postnatal exposures (postnatal days 4–7 and 10–13) to inhaled Fe oxide nanoparticles to determine the extent to which it contributed to UFP-based air pollution-related neurologic diseases and disorders. Fe (1.0 μg/m^3^) was inhaled either alone or in conjunction with SO_2_ (1.31 mg/m^3;^ FeS). Inhalation of FeS was premised on prior reports that Fe concentrations in ambient air correlate with sulfate content, based on sulfate’s ability to mobilize Fe from its oxide form (Ghio et al., 1999), and SO_2_ was found to increase Fe uptake into bronchial epithelium and altered its distribution among different cell types (Watson and Brain, 1980). Sulfur is critical to brain iron sulfur cluster proteins and critically involved in the brain transulfuration pathway (Vitvitsky et al., 2006). Moreover, studies of SO_2_ inhalation alone have been associated with ischemic stroke in humans (Wang et al., 2020; Szyszkowicz, 2008; Dong et al., 2018; Tian et al., 2018) and more rapid decline in cognitive function in Alzheimer’s disease (Lee et al., 2023), while experimental studies demonstrate its ability to cause brain injuries resembling cerebral ischemia in rats (Sang et al., 2010), to produce spatial memory impairments and alter glutamatergic function in mice (Yao et al., 2015), and lead to an imbalance of pro-/anti-inflammatory cytokines in brain of rats after combined PM2.5 and SO2 inhalation (Yang et al., 2017).

In our studies in mice, both Fe and FeS exposures altered brain metal correlations as determined at the termination of exposure (postnatal day 14) particularly in frontal cortex, and to a greater extent with FeS than Fe alone (Sobolewski et al., 2022). At postnatal day 14, alterations in frontal cortex and striatal neurotransmitter levels were found in response to FeS exposure that were opposite in direction in males (up) and females (down), while Fe alone had minimal effects. By postnatal day 60, however, many of these neurotransmitter changes seen in response to FeS were no longer evident (Sobolewski et al., 2022), suggestive of recovery of neurotransmitter function.

Nevertheless, despite the apparent recovery of neurotransmitter levels, subsequent behavioral tests revealed lingering deficits in parameters of responding on various operant schedules of food reward in both the Fe- and the FeS-exposed cohorts, suggestive of residual alterations in neurotransmitter function (Eckard et al., 2023). Consequently, the current study sought to ascertain whether behavioral experience unmasked residual neurotransmitter system alterations related to developmental exposures to Fe or FeS that would be consistent with long-term effects. For that purpose, brains from a subset of behaviorally-experienced and non-behaviorally-experienced male and female mice exposed to FeS or filtered air were collected at PND357 and those exposed to Fe or filtered air were collected at PND215 for assessment of changes in neurotransmitter trajectories.

## Materials and methods

### Animals

C57BL6/J female mice were bred monogamously with C57BL6/J male mice, with the latter removed following sperm plug identification. Mice were housed in standard mouse caging that included 1/8” high performance bedding (BioFresh, WA, United States), under a 12 h light-dark cycle, maintained at 22 ± 2°C and fed standard rodent chow at the University of Rochester Medical Center. Following birth, offspring were housed with the dam until weaning on postnatal day (PND) 21. After weaning, offspring were pair housed by sex and treatment group for the duration of the study. Subsets of offspring were euthanized at either postnatal day 215 (Fe) or 357 (FeS) and tissue harvested for various analyses. A single male and female pup from each litter were used for each endpoint so that no more than one pup/litter/sex was included. Sample sizes were *n* = 12 per sex/treatment group/behavioral condition for both the FeS and Fe cohorts.

The subset of offspring harvested at PND215 (Fe cohort) and PND357 (FeS cohort) had been divided into a behavior (B) and non-behavior (NB) groups. As previously described (Eckard et al., 2023), the behavioral subset within the Fe cohort had been screened for locomotor activity (PND70), novel object recognition (PND75), performance on a fixed interval (FI) 60 s schedule of food reward (PND110-142), performance on a differential reinforcement of low rate (DRL) schedule of food reward (PND143-180) and performance a fixed ratio waiting for reward schedule of food reinforcement (PND185-200). The behavioral subset within the FeS cohort had been screened for locomotor activity (PND65), novel object recognition (PND86), performance on a FI 60 s schedule of food reward (119–155), and performance on a DRL schedule of food reward (PND239-271); the behavioral subset from the FeS cohort was not tested on the fixed ratio waiting for reward schedules. For both the Fe and FeS cohorts, the NB subset remained in their home cages throughout this period of behavioral testing but were comparably food-restricted during the period of behavioral testing as the behaviorally-tested mice. The longer time trajectory for collection of brains from the FeS cohort as compared to the Fe cohort was due to the fact that breeding and exposures for this cohort preceded that for the Fe cohort.

All protocols and treatments used with mice were approved by the University of Rochester Medical Center Institutional Animal Care and Use, and in accordance with NIH guidelines.

### Fe and FeS exposures

Mice were exposed either to iron (Fe) or Fe and sulfur (FeS). Specifically, exposures were to Fe oxide (Fe) nanoparticles alone or with sulfur dioxide (SO_2_) gas. Exposures took place from PND 4–7 and 10–13 and were 4 h per day in duration. The exposures to Fe and to FeS were carried out at separate times, however each included a corresponding control group of mice exposed to filtered air at the same time. Exposures were carried out in whole body inhalation cages. The intended Fe concentration was 1.0 μg/m^3^ which was based on a range of values reported for outdoor Fe levels (Chen et al., 2017; Bem et al., 2003; Mansha et al., 2012), while the intended SO_2_ concentration was 1.31 mg/m^3^, as based on the U. S. Environmental Protection Agency secondary standard for SO_2_.

Ultrafine (UFP) particles of Fe-oxide were generated using electric spark discharge between two 99.99999% pure iron rods (3N5 Purity, ESPI Metals, Ashland, OR, United States) using a GFG-1000 Palas generator (Palas GmbH, Karlshrue, Germany). Particles were then fed into a compartmentalized whole-body mouse exposure chamber; HEPA-filtered air was delivered to a control chamber, consistent with prior studies in our inhalation facility (Oberdörster et al., 2000). Particle charge was brought to Boltzmann equilibrium by passing the airborne particles through a deionizer

(Model P-2031, NRD, Grand Island, NY, United States). For adjustment of particle number concentration, electric spark discharge frequency was altered. Aerosol number concentration and particle size were monitored in real-time using a Condensation Particle Counter (CPC, model 3022, TSI Inc., St Paul, MN, United States) and a Scanning Mobility Analyzer (SMPS, model 3934 TSI Inc., St Paul, MN, United States) respectively. To generate Fe-oxide particles, a low flow of oxygen (∼ 50 mL/min) was added into the argon flow (∼ 5 L/min) entering the spark discharge chamber. An O_2_ sensor (MAXO2 -250E, Maxtec, Salt Lake City, UT, United States) was used to verify an oxygen concentration of 21% in the exposure chamber. This procedure resulted in particle sizes that were exclusively in the ultrafine size range that exhibited a count median diameter (CMD) of approximately 12–14 nm. Mass concentrations of Fe were measured using ICP-OES analysis on nitrocellulose membrane filters (0.8 micron, AAWP02500, Millipore Ltd., Tullagreen, Cork, IRL) that were collected daily (5 L/min for 60 min., 300L total volume) from both the filtered air and ultrafine Fe-oxide particle exposure chambers. To generate concurrent SO_2_ exposures, SO_2_ compressed in gas cylinders (EPA Protocol Standard, 50 ppm, Airgas East, Radnor, PA, United States) was diluted with filtered air. It was then bled into (200 mL/min) the Fe-oxide containing conduit to achieve final desired concentrations for Fe + SO_2_ exposures. The SO_2_ concentrations were continuously monitored using an SO_2_ gas monitor (model 43C, Thermo Environmental Instruments Inc., Franklin, MA, United States). This Fe-oxide/SO_2_ mixture was fed into the whole-body exposure chamber at 25–30 L per minute.

### Brain neurotransmitter and trans-sulfuration analyses

As previously described (Sobolewski et al., 2022), neurotransmitter levels were quantified by the University of Rochester Mass Spectrometry

Core. Frontal cortex and striatum were diluted in 75 μL of ice-cold acetonitrile (50%, v/v) and subsequently homogenized for 10 s via ultrasonication (SLPe Digital Sonifier, Branson Ultrasonics Corp., Danbury, CT.), after which the homogenate was centrifuged at 10,000 g (4°C) for 20 min. The resulting supernatant was then centrifuged at 10,000 g (4°C) for 20 min, after which the new supernatant was collected and stored at –80°C until analysis. Stock solutions were made for neurotransmitters. A standard mixture was then made using ddH_2_O, and analyte concentrations varied based on prior range-finding studies, in order to accommodate region-specific variations in endogenous neurotransmitters. To create internal standards for each individual neurotransmitter, the stock solution was derivatized using 13C6 benzoyl chloride (BzCl, Sigma Aldrich) via a method adapted from Wong et al. (2016). The derivatized internal standard mixture was frozen at −80°C for long term storage. Prior to being added to the samples, internal standard aliquots were thawed, then diluted in 50% acetonitrile with 1% sulfuric acid. Samples were derivatized following the same procedure prior to analysis. Samples were first centrifuged at 16,000 g for 5 min to remove debris, after which 20 μL of supernatant was placed in a clean LoBind tube (Eppendorf). Subsequently, 10 μL of 100 mM sodium carbonate,10 μL of 2% BzCl in acetonitrile, and 10 μL of the respective internal standard was added in sequence. To reduce the organic concentration prior to injection, 50 μL of ddH_2_O was added. Samples were then centrifuged again to pellet any remaining protein before the supernatant was added to a clean autosampler vial. LC-MS/MS analysis was carried out using a Dionex Ultimate 3000 UHPLC that was coupled to a Q Exactive Plus mass spectrometer (Thermo Fisher). Analytes were separated on a Waters Acquity HSS T3 column. The mobile phases used were: A) 10 mM ammonium formate in 0.1% formic acid, and B) acetonitrile. The flow rate was set to 400 μL/min and the column oven was operated at 27°C. After 5 μL of each sample was injected, the analytes were separated using a 12 min multi-step gradient. The Q Exactive Plus was operated in positive mode, and included a parallel reaction monitoring method (PRM) to detect derivatized molecules. the LC Quan node of the XCalibur software (Thermo Fisher) was used to extract fragment ions with a 10 ppm mass error. Endogenous analyte peak

areas were compared to those of each internal standard to determine relative abundance. These values were then divided by wet weight of the sample.

### Statistical analyses

Outliers were first analyzed using a Grubb’s test (Prism Graph Pad), with no more than one outlier removed from any group, and the mean value of the remaining values was then substituted for a removed value. Data were subsequently analyzed via two factor ANOVAs with behavioral experience (BE) and Fe or FeS treatment (TX) as factors. *Post hoc t*-tests were performed if a significant interaction of behavior by treatment (BE × TX) was found in the ANOVA. Assessment of the impact of BE itself was determined by comparisons of B vs. NB air exposure groups. Effects of Fe and FeS under NB conditions involved comparisons of Fe and FeS NB groups with air NB groups. Effects of Fe or FeS under B conditions involved comparisons of Fe or FeS B groups with corresponding Air B groups. For the graphs, data were presented as a percent of the NB Air groups within each cohort. *P*-values of ≤ 0.05 were considered statistically significant, and some *p*-values ≤ 0.10 are reported where relevant.

## Results

### Effects of behavioral experience on neurotransmitter and trans-sulfuration levels

Table 1 summarizes changes in neurotransmitter levels and trans-sulfuration markers in frontal cortex and striatum in relation to behavioral experience in both the FeS and Fe exposure conditions based on comparisons of NB air groups to B air groups. As it shows, changes were far more prominent in males, and were found in both brain regions and in both FeS and Fe exposure cohorts. Of note, consistent increases in serotonin precursors tryptophan and kynurenine were found across sexes and Fe exposures.

**TABLE 1:** Summary of effects of behavioral experience.

	Females				Males			
	FC/FeS	FC/Fe	STR/FeS	STR/Fe	FC/FeS	FC/Fe	STR/FeS	STR/Fe
**Neurotransmitter**								
Glutamine					↑	↑	↑	
Glutamate					↑	↑	↑	
GABA					∼↑	↑	↑	
Gln/Glu								
Glu/GABA						∼↑		

Tryptophan	↑	↑	↑	↑	↑	↑	↑	↑
Kynurenine	↑	↑	↑	↑	↑	↑	↑	↑
5HTP					↑		↑	
5HT					↑	↑	↑	
5HIAA					↑	↑	↑	
5HIAA/5HT					↑	∼↑	↑	

Tyrosine					↑	∼↑	↑	
HVA							↑	
DOPAC					∼↑		↑	
DA		↑						
NE								
HVA/DA	↑	↓					↑	
DOPAC/DA		↓					↑	

GSH					↑	↑	↑	
H-Cysteine	↑	↑		↑	↑		↑	↑
Cysteine					∼↑		↑	
Methionine							↑	

#### Females

**FIGURE 1:**
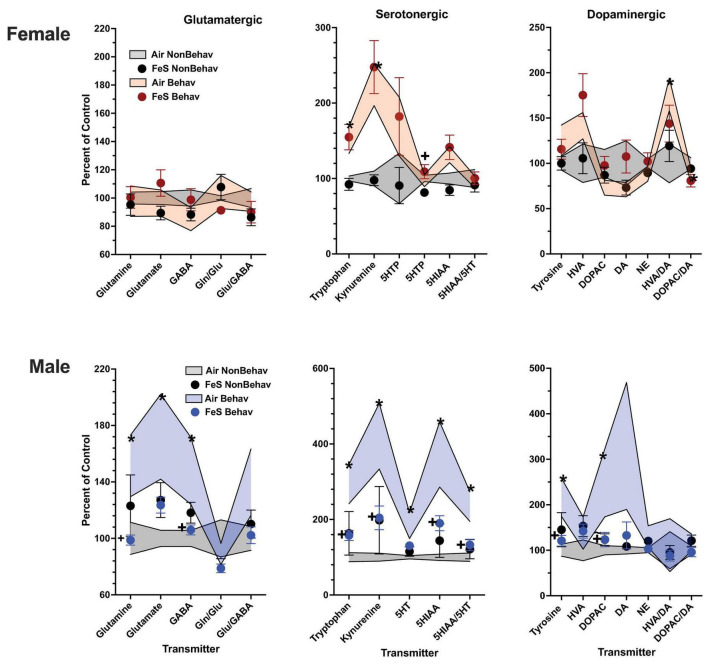
Group mean ± S.E. value of glutamatergic, serotonergic and dopaminergic neurotransmitter levels in frontal cortex for females (top row) and males (bottom row). All data are plotted as percent of Air non-behavior group values: gray shading = air non-behavior control; blue shading = air behavior control; black circles = FeS exposed non-behavioral group; pink circles = female FeS behavioral exposed group; blue circles = male FeS behavioral exposed group. * = significant difference between air behavioral group and air non-behavior group values; += significant difference between FeS group and corresponding air control. Gln/Glu, glutamine/glutamate; Glu/GABA, glutamate/GABA; HVA, homovanillic acid; DOPAC, 3,4-dihydroxyphenylacetic acid; DA, dopamine; NE, norepinephrine; 5HT, serotonin; 5HIAA, 5-hydroxyindoleacetic acid.

**FIGURE 2:**
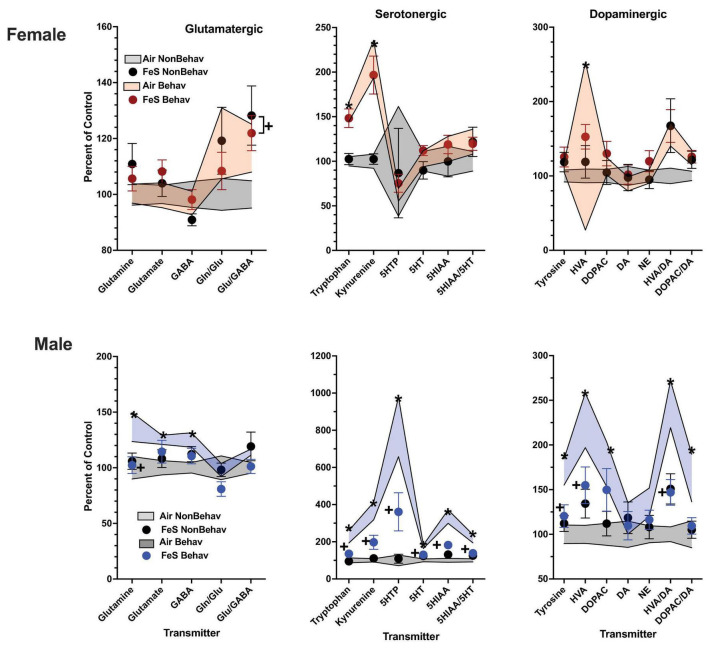
Group mean ± S.E. value of glutamatergic, serotonergic and dopaminergic neurotransmitter levels in striatum for females (top row) and males (bottom row). All data are plotted as percent of Air non-behavior group values: gray shading = air non-behavior control; blue shading = air behavior control; black circles = FeS exposed non-behavioral group; pink circles = female FeS behavioral exposed group; blue circles = male FeS behavioral exposed group. * = significant difference between air behavioral group and air non-behavior group values; += significant difference between FeS group and corresponding air control. Gln/Glu, glutamine/glutamate; Glu/GABA, glutamate/GABA; HVA, homovanillic acid; DOPAC, 3,4-dihydroxyphenylacetic acid; DA, dopamine; NE, norepinephrine; 5HT, serotonin; 5HIAA, 5-hydroxyindoleacetic acid.

In females, behavioral experience increased levels of tryptophan and kynurenine in both brain regions and Fe conditions (Figure 1; Table 1; Supplementary Table 1). Specifically, within the FeS cohort, behavioral experience *per se* significantly increased levels of frontal cortical tryptophan (+ 151%), kynurenine (+ 224%), and HVA/DA (+ 176%) and of striatal (Figure 2; Table 1; Supplementary Table 1) tryptophan (+ 155%) and kynurenine (+ 215%).

**FIGURE 3:**
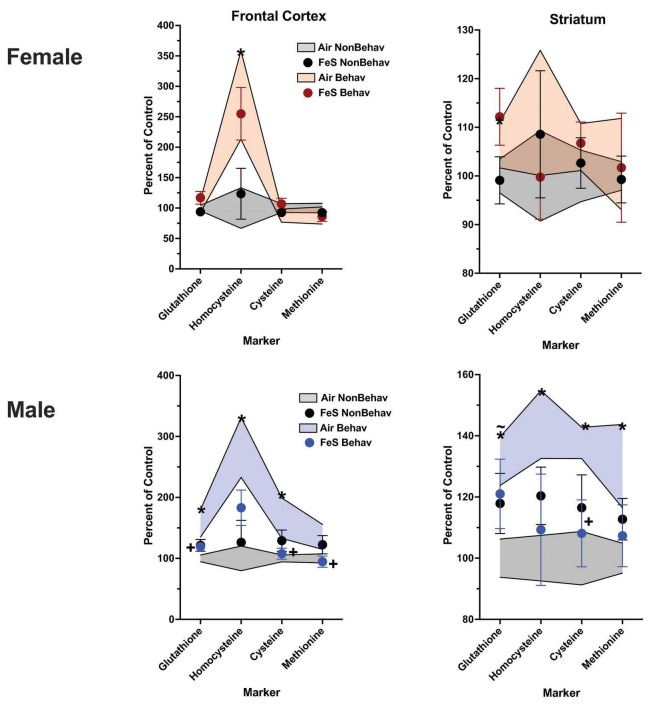
Group mean ± S.E. value of trans-sulfuration markers for females (top row) and males (bottom row) in frontal cortex (left column) and striatum (right column). All data are plotted as percent of Air non-behavior group values: gray shading = air non-behavior control; blue shading = air behavior control; black circles = FeS exposed non-behavioral group; pink circles = female FeS behavioral exposed group; blue circles = male FeS behavioral exposed group. * = significant difference between air behavioral group and air non-behavior group values; += significant difference between FeS group and corresponding air control; ^∼^* = marginally significant difference between air behavioral group and air non-behavioral group.

With respect to trans-sulfuration markers, behavioral experience significantly elevated levels of frontal cortical homocysteine in females (+ 285%, Figure 3; Table 1; Supplementary Table 1).

**FIGURE 4:**
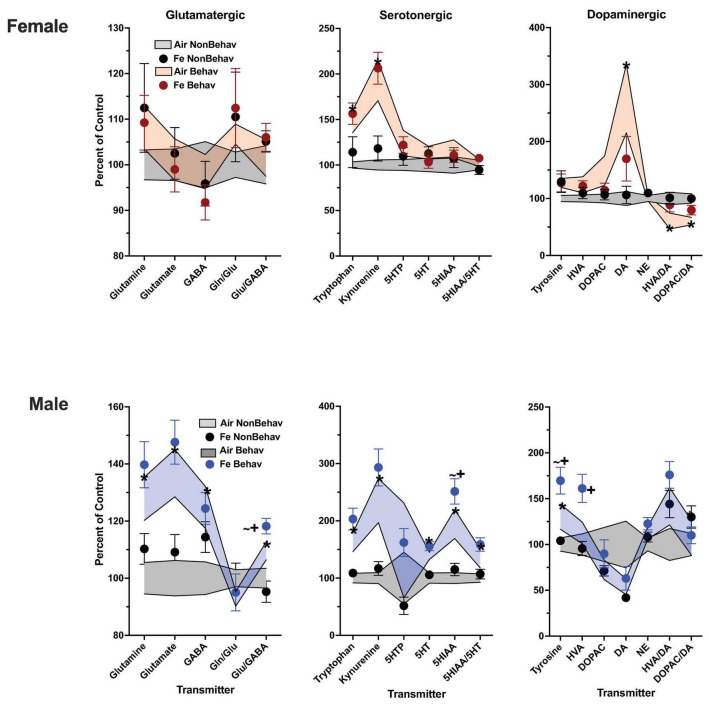
Group mean ± S.E. value of glutamatergic, serotonergic and dopaminergic neurotransmitter levels in frontal cortex for females (top row) and males (bottom row). All data are plotted as percent of Air non-behavior group values: gray shading = air non-behavior control; blue shading = air behavior control; black circles = Fe exposed non-behavioral group; pink circles = female Fe behavioral exposed group; blue circles = male Fe behavioral exposed group. * = significant difference between air behavioral group and air non-behavior group values; += significant difference between Fe group and corresponding air control; ∼+ = marginally significant difference between Fe group and corresponding air control. Gln/Glu, glutamine/glutamate; Glu/GABA, glutamate/GABA; HVA, homovanillic acid; DOPAC, 3,4-dihydroxyphenylacetic acid; DA, dopamine; NE, norepinephrine; 5HT, serotonin; 5HIAA, 5-hydroxyindoleacetic acid.

**FIGURE 5:**
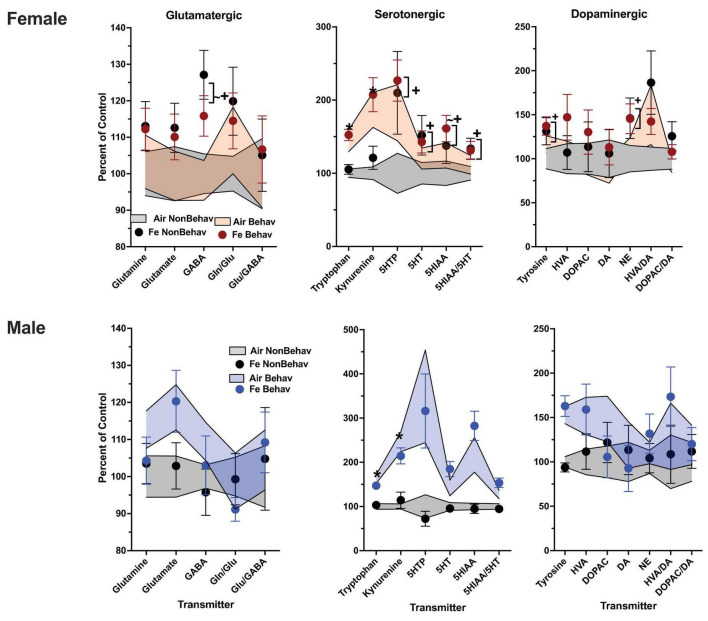
Group mean ± S.E. value of glutamatergic, serotonergic and dopaminergic neurotransmitter levels in striatum for females (top row) and males (bottom row). All data are plotted as percent of Air non-behavior group values: gray shading = air non-behavior control; blue shading = air behavior control; black circles = Fe exposed non-behavioral group; pink circles = female Fe behavioral exposed group; blue circles = male Fe behavioral exposed group. * = significant difference between air behavioral group and air non-behavior group values; += significant difference between Fe group and corresponding air control; ∼+ = marginally significant difference between Fe group and corresponding air control. Gln/Glu, glutamine/glutamate; Glu/GABA, glutamate/GABA; HVA, homovanillic acid; DOPAC, 3,4-dihydroxyphenylacetic acid; DA, dopamine; NE, norepinephrine; 5HT, serotonin; 5HIAA, 5-hydroxyindoleacetic acid.

In the Fe cohort, behavioral experience-produced increases in both frontal cortical and striatal tryptophan (+ 147% and + 144%, respectively) and kynurenine (+ 193% and + 186%, respectively) (Figures 4, 5; Table 1; Supplementary Table 1). Frontal cortical dopaminergic system changes also included increases in dopamine (+ 275%) as well as reductions in levels of dopamine turnover (HVA/DA = -39%, DOPAC/DA = −40%).

**FIGURE 6:**
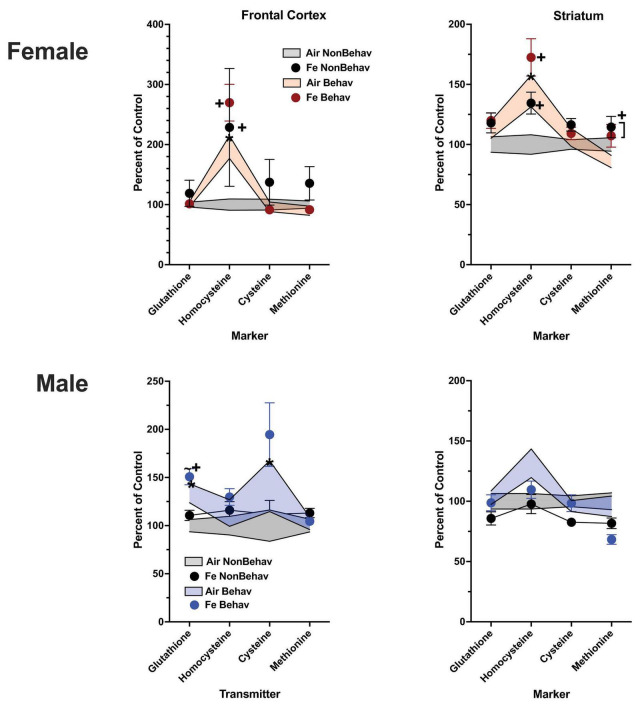
Group mean ± S.E. value of trans-sulfuration markers for females (top row) and males (bottom row) in frontal cortex (left column) and striatum (right column). All data are plotted as percent of Air non-behavior group values: gray shading = air non-behavior control; blue shading = air behavior control; black circles = Fe exposed non-behavioral group; pink circles = female Fe behavioral exposed group; blue circles = male Fe behavioral exposed group. * = significant difference between air behavioral group and air non-behavior group values; += significant difference between Fe group and corresponding air control; ∼+ = marginally significant difference between Fe group and corresponding air control.

With respect to trans-sulfuration markers, increases in both FeS frontal cortical (+ 196%) and Fe striatal (+ 144%) homocysteine levels were also found in females (Figure 6; Table 1; Supplementary Table 1).

#### Males

In the case of males, behavioral experience-induced alterations in neurotransmitter levels were far more extensive. In the FeS exposures, frontal cortical changes (Figure 1; Table 1; Supplementary Table 1) included elevations in levels of glutamine, glutamate and GABA (+151, +172, +148%, respectively), all markers of serotonergic function (tryptophan: +294%, kynurenine: +421%, 5HT: +184%, 5HIAA: +372%, 5HIAA/5HT: +234%) as well as markers of dopaminergic function (tyrosine: +216%, DOPAC: +248%). Similar findings were found in striatum (Figure 2; Table 2; Supplementary Table 1), glutamine: +137%, glutamate: +125%, GABA: +125%, tryptophan: +227%, kynurenine: +365%, 5HTP: +818%, 5HT: +173%, 5HIAA: +335%, 5HIAA/5HT: + 216%; tyrosine: + 170%, HVA: + 228%, DOPAC: + 178%, HVA/DA: + 246%, DOPAC/DA: + 163%).

**TABLE 2:** Effects of FeS and Fe exposure effects in frontal cortex and striatum in relation to behavioral and non-behavioral condition.

	Female								Male							
	Frontal cortex				Striatum				Frontal cortex				Striatum			
	FeS		Fe		FeS		Fe		FeS		Fe		FeS		Fe	
Neurotransmitter	NB	B	NB	B	NB	B	NB	B	NB	B	NB	B	NB	B	NB	B
Glutamine										↓				↓		
Glutamate																
GABA							↑	↑		↓						
Gln/Glu																
Glu/GABA					↑	↑						∼↑				

Tryptophan										↓				↓		
Kynurenine										↓				↓		
5HTP							↑	↑						↓		
5HT							↑	↑		↓				↓		
5HIAA							↑	↑		↓		∼↑		↓		
5HIAA/5HT							↑	↑						↓		

Tyrosine							↑	↑		↓		∼↑		↓		
HVA												↑		↓		
DOPAC										↓						
DA																
NE							↑	↑								
HVA/DA														↓		
DOPAC/DA																

GSH										↓		∼↑				
H-Cysteine			↑	↑			↑	↑								
Cysteine										↓				↓		
Methionine							↑	↑		↓						

Levels of markers of the trans-sulfuration pathway were also increased in response to behavioral experience in males in frontal cortex (Figure 3; Table 1; Supplementary Table 1), glutathione (+ 137%, homocysteine: + 282%, cysteine: + 166%) and in striatum (glutathione: + 131%, homocysteine: + 143%, cysteine: + 137%, methionine: + 131%).

In the Fe cohort, behavioral experienced increased levels of neurotransmitters in males primarily in frontal cortex (Figure 4; Table 1; Supplementary Table 1), including levels of glutamine (+ 127%), glutamate (+ 137%), GABA (+ 125%), glutamate/GABA (+ 110%), tryptophan (+ 162%), kynurenine (+ 235%), 5HT (+ 139%), 5HIAA (+ 194%), 5HIAA/5HT (+ 134%), and tyrosine (+ 130%). Increases were also seen in frontal cortical glutathione levels (+ 134%).

Increases in striatum in response to behavioral experience (Figure 5; Table 1; Supplementary Table 1) were limited to tryptophan (+ 161%) and kynurenine (+ 246%).

Increases in striatal trans-sulfuration markers (Figure 6; Table 1; Supplementary Table 1) were found only for homocysteine (+ 142%).

### Effects of FeS on neurotransmitter and trans-sulfuration levels

#### Non-behavioral condition

Under non-behavioral conditions, FeS exposures were not associated with any changes in frontal cortex neurotransmitters in females or in males (Figure 1; Table 2). In striatum (Figure 2; Table 2), however, increases in levels of excitotoxicity (glutamate/GABA) were found in FeS females that actually included both the NB and B conditions [Glu/GABA, + 117%, TX *F*(3, 44) = 2.13, *p* = 0.039]. No changes in response to FeS under NB conditions were seen in male striatum.

Developmental FeS exposure had no impact on frontal cortical (Figure 3; Table 2) or striatal trans-sulfuration markers in either sex under NB conditions.

#### Behavioral condition

No changes were found in response to FeS (Figures 1, 2; Table 2) under behavioral conditions in female frontal cortex or in striatum other than the increased exicitotoxicity that was seen across behavioral conditions. In contrast, FeS exposure in males (Figure 1; Table 2) significantly precluded the behavioral experience-associated increases in brain neurotransmitter levels in frontal cortex. This is shown by the lack of change in neurotransmitter levels in the FeS B group as compared to NB air, as well as the reductions in levels in the FeS B group as compared to the B Air group. Specifically, significant reductions were found within the FeS B group relative to FeS air B controls in frontal cortical levels of glutamine [BE × TX *F*(3, 42) = 2.27, *p* = 0.0029; B Air vs. B FeS p = 0.031], GABA [BE × TX *F*(3,42) = 2.25, *p* = 0.03; B Air vs. B FeS *p* = 0.032], tryptophan [BE × TX *F*(3, 42) = 2.5, *p* = 0.016, B Air vs. B FeS *p* = 0.02], kynurenine [BE × TX *F*(3, 42) = 2.44, *p* = 0.0192, B Air vs. B FeS *p* = 0.023], 5HIAA [BE × TX *F*(3, 42) = 2.21, *p* = 0.032, B Air vs. B FeS *p* = 0.0156), 5HIAA/5HT [BE × TX *F*(3, 42) = 2.4, *p* = 0.021, B Air vs. B FeS *p* = 0.0076], tyrosine [BE × TX *F*(3, 42) = 2.36, *p* = 0.023, B Air vs. B FeS *p* = 0.029] and marginally of DOPAC [BE × TX *F*(3, 42) = 1.82, *p* = 0.076, B Air vs. B FeS *p* = 0.036].

As was the case in male frontal cortex, the increases in striatal glutamatergic, serotonergic and dopaminergic neurotransmitter levels seen in response to behavioral experience were precluded by FeS exposures (Figure 2; Table 2), with reductions in FeS exposed males from B groups found in levels of glutamine [BE × TX *F*(3, 41) = 2.04, *p* = 0.048, B Air vs. B FeS *p* = 0.022], tryptophan [BE × TX *F*(3, 41) = 2.23, *p* = 0.032, B Air vs. B FeS *p* = 0.0025], kynurenine [BE × TX *F*(3, 41) = 3.00, *p* = 0.0046, B Air vs. B FeS *p* = 0.0004], 5HTP [BE × TX *F*(3, 41) = 2.5, *p* = 0.016, B Air vs. B FeS *p* = 0.0017], 5HT [BE × TX *F*(3, 41) = 3.78, *p* = 0.0005, B Air vs. B FeS *p* = 0.0018], 5HIAA [BE × TX *F*(3, 41) = 4.54, *p* < 0.0001, B Air vs. B FeS *p* < 0.0001], 5HIAA/5HT [BE × TX *F*(3, 41) = 3.83, *p* = 0.0004, B Air vs. B FeS *p* = 0.0003], tyrosine [BE × TX *F*(3, 41) = 2.64, *p* = 0.0118, B Air vs. B FeS *p* = 0.006], HVA [BE × TX *F*(3, 41) = 2.64, *p* = 0.0118, B Air vs. B FeS *p* = 0.0186] and HVA/DA [BE × TX *F*(3, 42) = 4.25, *p* = 0.0001, B Air vs. B FeS *p* = 0.0004] relative to B air controls.

In FeS exposed males subjected to behavioral experience, significant reductions in trans-sulfuration markers were observed relative to behavior air males, as was the case with neurotransmitters (Figure 3; Table 2). This included decrements in frontal cortex glutathione [BE × TX *F*(3, 42) = 2.25, *p* = 0.0296, B air vs. B FeS *p* = 0.0476], cysteine [BE × TX *F*(3, 42) = 2.18, *p* = 0.0347, B air vs. B FeS *p* = 0.0457] and methionine [BE × TX *F*(3,42) = 2.23, *p* = 0.0315] and in striatal cysteine [BE × TX *F*(3, 42) = 2.5, *p* = 0.0165, B air vs. B FeS *p* = 0.0338].

### Effect of Fe on neurotransmitter and trans-sulfuration levels

#### Non-behavioral condition

Changes in response to Fe only exposure under NB conditions were not seen in frontal cortex in either males or females (Figure 4; Table 2) in glutamatergic, serotonergic or dopaminergic neurotransmitter levels.

In female striatum (Figure 5; Table 2), Fe increased levels of GABA [TX *F*(3, 42) = 3.88, *p* = 0.0004, NB Air vs. NB Fe *p* = 0.0023] within the glutamatergic class of neurotransmitters, 5HTP [TX *F*(3, 42) = 2.04, *p* = 0.048], 5HT [TX *F*(3, 42) = 2.09, *p* = 0.0423], marginally increased 5HIAA [TX *F*(3, 42) = 1.97, *p* = 0.0552], and significantly increased 5HIAA/5HT [TX *F*(3, 42) = 2.19, *p* = 0.0338] within the serotonergic class, as well as tyrosine [TX *F*(3, 42) = 2.23, *p* = 0.031], and NE [TX *F*(3, 42) = 2.36, *p* = 0.023] within the dopaminergic class of neurotransmitters under NB conditions. In contrast, Fe had no influence in male striatum.

In the Fe cohort (Figure 6; Table 2), levels of homocysteine were increased by Fe in females in frontal cortex [TX *F*(3, 43) = 2.14, *p* = 0.0382] and striatum [TX *F*(3, 42) = 2.58, *p* = 0.0136], as were levels of striatal methionine [TX *F*(3, 42) = 2.42, *p* = 0.0199]. In contrast, no effects of Fe were seen in in male frontal cortex or striatum.

#### Behavioral condition

Frontal cortical neurotransmitter levels were not significantly impacted by Fe exposure in females (Figure 4; Table 2). In the behavioral experience condition, males exposed to Fe exhibited a marginal increase in levels of frontal cortical Glu/GABA (B Air vs. B Fe *p* = 0.0255), 5HIAA (B Air vs. B Fe *p* = 0.0225) and tyrosine (B Air vs. B Fe *p* = 0.0149), and significant increases in HVA [BE × TX *F*(3, 44) = 2.09, *p* = 0.043, B Air vs. B Fe *p* = 0.0072] relative to B air controls.

In striatum of females (Figure 5; Table 2), Fe exposure increased levels of GABA [TX *F*(3, 42) = 3.88, *p* = 0.0004, NB Air vs. NB Fe *p* = 0.0023], 5HTP [TX *F*(3, 42) = 2.04, *p* = 0.048], 5HT [TX *F*(3, 42) = 2.09, *p* = 0.0423], marginally increased 5HIAA [TX *F*(3, 42) = 1.97, *p* = 0.0552], and significantly increased 5HIAA/5HT [TX *F*(3, 42) = 2.19, *p* = 0.0338], tyrosine [TX *F*(3, 42) = 2.23, *p* = 0.031], and NE [TX *F*(3, 42] = 2.36, *p* = 0.023 across NB and B conditions. In contrast, neurotransmitter levels in male striatum were not influenced by Fe exposures.

In the case of Fe only exposure (Figure 6; Table 2), levels of homocysteine were increased by Fe in females in frontal cortex [TX *F*(3, 43) = 2.14, *p* = 0.0382] and striatum [TX *F*(3, 42) = 2.58, *p* = 0.0136], as were levels of striatal methionine [TX *F*(3, 42) = 2.42, *p* = 0.0199] under conditions of behavioral experience. In contrast, no effects of Fe vs. air were seen in either in male frontal cortex or striatum.

## Discussion

While the impact of Fe deficiency on brain development has been extensively investigated, little is as yet known about the consequences of early elevated brain Fe, despite the fact that Fe is one of the most abundant metals in air pollution and exposures to AP are life-long, with the potential for direct olfactory uptake of ultrafine particulate matter AP with Fe contamination (Oberdörster et al., 2004). Correspondingly, in our prior studies, developmental exposure to Fe or FeS nanoparticle inhalation was found to result in alterations in brain neurotransmitter levels at postnatal day 14, i.e., 24 hr. following termination of such exposures, that included elevations in frontal cortical and striatal glutamatergic and dopaminergic neurotransmitters that were male-biased and more pronounced and selective to FeS exposures (Sobolewski et al., 2022). However, these effects were not seen by PND 60, suggesting reversibility of effects. The fact that behavioral assessments subsequent to PND60 revealed changes in operant behavior prompted the current study (Eckard et al., 2023), as these findings suggested that behavioral experience, an inevitable component of human life, had unmasked residual and protracted consequences of developmental whole body inhalational exposures to ultrafine Fe alone or Fe with SO_2_ (FeS) on brain neurotransmitter functions that subserve these behavioral functions.

In accord with that hypothesis, the current study revealed two major effects. First, while behavioral experience dramatically increased levels of brain neurotransmitters, as would be expected given plasticity of brain (Spitzer, 2015; Hernández et al., 2011; Meeusen and De Meirleir, 1995; Froemke, 2015), these increases were precluded by prior developmental exposures to FeS, but not Fe only, in a male-biased fashion, particularly for brain serotonergic function. In addition, females showed increased striatal levels of neurotransmitters, particularly in serotonergic function and in homocysteine, in response to prior developmental Fe only exposure, with these latent consequences occurring regardless of behavioral experience condition.

As noted above, behavioral experience itself markedly increased neurotransmitter levels within glutamatergic, serotonergic and dopaminergic neurotransmitter classes in both frontal cortex and striatum, as well as brain markers of trans-sulfuration, as seen in comparisons of Air NB vs. Air B groups. This increase was highly male-biased and seen in both the FeS and Fe cohorts. The prior behavioral experience in this study consisted of protracted exposures to positive schedules of food reward, including both FI and DRL schedules that were followed in both the FeS and Fe cohorts by marked increases in brain neurotransmitter levels. Such increases are consistent with long-held knowledge of the underlying neurochemical mediation of rewarded behavior (Wise and McDevitt, 2018; Vlachou and Markou, 2010; Macoveanu, 2014; Lajtha, 2008). These increases were particularly notable in males and in serotonergic and dopaminergic function in both frontal cortex and striatum, where females evidenced increases in the precursors of 5HT, namely tryptophan and kynurenine, and males showed increases in precursors as well as serotonin, its metabolite and turnover. Such findings are consistent with a body of work that has documented that sustained increases can occur in both dopamine and serotonin levels in response to behaviors including learning, and that these can change the subsequent reactions of dopamine and serotonin neurons (Yagishita, 2020).

The fact that both serotonergic and dopaminergic classes of neurotransmitters showed the most pronounced increases in response to behavioral experience may likely reflect their interactive effects (Fischer and Ullsperger, 2017). More specifically, serotonergic and dopaminergic systems have been found to be involved in behaviors such as the DRL and FI schedules of reinforcement and thus increases in behaviorally-trained air exposed mice would be expected. For example, serotonin depletion in the nucleus accumbens was found to affect inhibitory control on a DRL schedule of reinforcement (Fletcher et al., 2009). Similarly, administration of selective 5-HT reuptake inhibitors were found to decrease efficiency of responding on a DRL schedule (Dekeyne et al., 2002). Dopaminergic dysfunction likewise affects efficiency of DRL responding (Neill and Herndon, 1978), including dopamine depletion in the medial prefrontal cortex in the rat (Sokolowski and Salamone, 1994). Our prior studies have shown the critical role of dorsomedial striatal dopaminergic mediation of FI schedule-controlled behavior (Cory-Slechta et al., 2002; Cory-Slechta et al., 1998; Evans and Cory-Slechta, 2000). In addition, serotonergic compounds have been found to influence FI performance (Brady and Barrett, 1985). As such, the failure of FeS-exposed male mice to elevate brain neurotransmitter levels in contrast to what was seen in air-exposed brains may also underlie the alterations in both DRL and FI performance that were associated with these developmental exposures as described in our previous studies (Eckard et al., 2023), as such behaviors are dependent upon such elevations (Yagishita, 2020).

Another notable observation was that Fe alone induced increases in striatal serotonin and its turnover and in both frontal cortical and in striatal levels of homocysteine in females, effects that were independent of behavioral experience. There were no indications of such effects at either PND14 or PND60 (Sobolewski et al., 2022). Changes in serotonergic function have long been linked to depression (Parker and Brotchie, 2010), and reductions in serotonergic transmission and alterations in serotonin receptors constitute one of the earliest pathological changes in the prodromal phase of Alzheimer’s disease; both depression and Alzheimer’s disease are female-biased (Babić Leko et al., 2021; Kolahchi et al., 2024). The potential importance of such effects is further underscored by the fact that serotonin regulates the release of norepinephrine, dopamine and acetylecholine (Babić Leko et al., 2021). With respect to brain homocysteine levels, elevated serum homocysteine levels have long been implicated in Alzheimer’s disease (Gao et al., 2023), but whether elevated brain homocysteine levels contribute to Alzheimer’s pathology is less clear (Serot et al., 2005; Smach et al., 2011; Oikonomidi et al., 2016); serum levels of homocysteine were not examined here. Nevertheless, homocysteine is considered to be redox active and to alter redox homeostasis (Zou and Banerjee, 2005) and studies in a mouse model based on dietary induction reported that high levels of brain homocysteine enhanced tau pathology (Di Meco et al., 2019).

Collectively these protracted adverse consequences raise the obvious question as to how such effects might occur, since exposures to Fe or FeS in the current study occurred early in development, specifically from postnatal days 4–7 and 10–13 (Sobolewski et al., 2022) making it seem unlikely that elevated brain Fe was present at this far later stage of the lifecycle. However, in the face of potential brain metal dyshomeostasis, as produced by these exposures (Sobolewski et al., 2022), particularly FeS, as well as the insufficiency of mechanisms to remove Fe from brain and its lifelong accumulation, it may be that exposures lead to a sustained dysfunctional brain cellular Fe metabolism.

These protracted neurotransmitter changes seen here, as unmasked by behavioral experience, could also reflect enduring changes, such as epigenetic effects rendered earlier in development, or epigenetic changes induced by behavioral experience, or both. Acute effects of such exposures could include interactions of Fe with tryptophan hydroxylase and tyrosine hydroxylase. Tryptophan hydroxylase catalyzes the hydroxylation of L-tryptophan to 5-HTP which is then converted to serotonin (5-HT). Fe is a requisite cofactor for tryptophan hydroxylase catalysis, thereby setting the stage for potential interactions of FeS exposures with 5-HT production (Kuhn and Hasegawa, 2020). Similarly, ferrous iron is a cofactor for tyrosine hydroxylase, which catalyzes the conversion of L-tyrosine to L-dopa, the rate limiting step in the synthesis of the catecholamines such as dopamine (Frantom et al., 2006), such that FeS could also interact with dopamine synthesis. Studies have shown that administration of iron chelators can inhibit tyrosine hydroxylase and tryptophan hydroxylase.

Acute interactions of FeS with tryptophan hydroxylase and tyrosine hydroxylase could then have been followed by potential epigenetic changes. The ability of Fe exposures to modify epigenetic regulation has been described, in particular a role for labile Fe. In the case of brain Fe overload, FeII can enter cells via the divalent metal transporter 1 (DNMT1) (Anderson and Frazer, 2017). Two critical classes of epigenetic regulators actually require FeII. One is the ten-eleven translocation enzyme class (TET1/2/3) that is responsible for catalyzing the oxidation of 5-methylcytosine (5 mc), the mark of silenced transcription, into 5 hydroxymethylcytosine (5 hmc). Importantly, the latter is considered a stable epigenetic mark and thus might underlie long-term changes.

The block of neurotransmitter increases occurred in response to FeS exposure, but not to Fe alone. This raises two additional potential considerations. It may have been the case that FeS exposure resulted in higher initial brain Fe concentrations and thus greater impact. Such a possibility is suggested by the interesting report demonstrating that Fe_2_O_3_ uptake into bronchial epithelium was actually increased in the presence of SO_2_ in comparison to uptake in response to Fe_2_O_3_ alone (Watson and Brain, 1980). Whether such effects occur in nasal epithelium and ultimately in brain remains to be determined, and, if so, to what extent does this influence neurotoxicity, as Fe brain measurements were not determined here. Another potential basis for the failure to observe behavioral-experience related increases in brain neurotransmitter levels could be early Fe-induced ferroptosis and consequent neuronal loss. Ferroptosis is an Fe-dependent form of programed cell death as distinct from other forms, involving phospholipid peroxidation (Ma et al., 2024). It may be that ferroptosis-related neuronal loss has occurred, but the impact on brain neurotransmitter systems is not evident until the behavioral experience-requisite plasticity is invoked.

Another possibility that could occur, and potentially explain the enhanced neurotoxicity of FeS, is the interaction of SO_2_ itself with brain, as it has likewise been shown to induce neurotoxicity. At high exposure concentrations (7.00 ± 0.78, 14.00 ± 0.59, and 28.00 ± 3.56 mg/m^3^), inhalation of SO_2_ for 90 days in rats was found to impair spatial memory retention in a water maze and to reduce glutamate receptor subunits (Sang et al., 2010) and to produce differential effects on markers of synaptic plasticity dependent upon duration of exposure (Yao et al., 2016). SO_2_ inhalation can produce ischemic injury in hippocampal neurons in rats (Yun et al., 2013) as well as apoptosis in hippocampal neurons (Sang et al., 2009). Specific effects on brain neurotransmitter systems, however, are as yet unknown. Developmental FeS exposure in males also prevented the increases in levels of the trans-sulfuration system markers in both frontal cortex and striatum. Notably, SO_2_ inhalation has also been reported to alter glutathione levels specifically in male brain at various exposure concentrations (Geng and Meng, 2003; Meng and Zhang, 2003).Certainly additional studies of SO_2_ inhalation alone as well as with Fe_2_O_3_ will be required to ascertain respective impacts on brain neurotransmitters as well as on the brain trans-sulfuration pathways.

A limitation of the current findings is the difference in the time post-exposure at which brains were collected in the FeS (PND357) vs. the Fe only (PND215) cohorts, as the timing of the collection was only 15 days post-exposure for Fe only exposures, whereas it was a total of 86 days for the FeS cohort. This difference clearly limits direct comparisons between the two cohorts. Nevertheless, the collective findings indicate that either Fe alone, or Fe in conjunction with SO_2_ exposures can adversely impact brain neurotransmitter systems and trans-sulfuration pathways. Considered collectively, developmental exposures to either Fe or FeS resulted in behavioral impairments, and in both cases these tended to be male-biased (Eckard et al., 2023), both produced changes in frontal cortical and striatal neurotransmitter systems at PND14 that tended to resolve by PND60. However, as shown here, despite this apparent resolution, neurotransmitter changes and alterations in trans-sulfuration pathways were found long after PND60 in response to either Fe or FeS. Whereas in females, these latent alterations occurred after developmental Fe only exposure regardless of behavioral experience and were predominantly found in striatal serotonergic systems, in males, these changes were found only in behaviorally-experienced mice primarily in response to FeS as a lack of elevation across all three classes of neurotransmitters neurotransmitter levels as seen with behavioral experience itself.

Importantly, these findings suggest the potential effects of Fe-related exposures to be unmasked across time by environmental contexts/conditions. As such, these findings may be particularly relevant to the later in life neurodegenerative diseases and disorders now increasingly associated with air pollution exposures (Cory-Slechta et al., 2023; Fu and Yung, 2020; Hu et al., 2019; Gong et al., 2023). Further studies are needed to assist in understanding the mechanisms of these latent neurotransmitter changes and how they may lead to subsequent vulnerability to neurodegenerative diseases and disorders. These findings also underscore both the need for assessment of persistence of adverse developmental effects on brain and how they may be unmasked, as well as to the need for understanding how various components of air pollution influence brain, as this is critical to regulatory decisions for public health protection.

## Data Availability

The raw data supporting the conclusions of this article will be made available by the authors, upon reasonable request.
