# Supplementary material for: The protracted neurotoxic consequences in mice of developmental exposures to inhaled iron nanoparticles alone or in combination with SO2
Source: Front Behav Neurosci. 2025 Jul 25;19:1544974. doi: 10.3389/fnbeh.2025.1544974 (PMC12331690; doi:10.3389/fnbeh.2025.1544974)
Supplement: Supplementary file 1 [file Data_Sheet_1.docx]

**Supplementary Table 1**

**Statistical Outcomes for Behavioral Experience**

| **Female FC FeS** |  |  |  |
| --- | --- | --- | --- |
|  | Tryptophan |  | F(1,22)=8.05, p=0.0096 |
|  | Kynurenine |  | F(1,22)=17.9, p=0.0003 |
|  | HVA/DA |  | F(1,22)=7.3, p=0.013 |
|  | H-Cysteine |  | F(1,22)=5.41, p=0.0297 |
|  |  |  |  |
| **Female STR FeS** |  |  |  |
|  | Tryptophan |  | F(1,22)=20.79, p=0.0002 |
|  | Kynurenine |  | F(1,22)=24.48, p<0.0001 |
|  |  |  |  |
| **Females FC Fe** |  |  |  |
|  | Tryptophan |  | F(1,22)=14.33, p=0.001 |
|  | Kynurenine |  | F(1,22)=16.4, p=0.0005 |
|  | DA |  | F(1,22)=8.15, p=0.0092 |
|  | HVA/DA |  | F(1,22)=5.06, p=0.0034 |
|  | DOPAC/DA |  | F(1,22)=13.67, p=0.0013 |
|  | H-Cysteine |  | F(1,22)=20.51, p=0.0002 |
|  |  |  |  |
| **Females STR Fe** |  |  |  |
|  | Tryptophan |  | F(1,22)=8.78, p=0.0072 |
|  | Kynurenine |  | F(1,22)=12.32, p=0.002 |
|  | H-Cysteine |  | F(1,22)=7.2, p=0.0136 |
|  |  |  |  |
| **Males FC FeS** |  |  |  |
|  | Glutamine |  | F(1,22)=4.36, p=0.0487 |
|  | Glutamate |  | F(1,22)=5.54, p=0.0279 |
|  | GABA |  | F(1,22)=3.87, p=0.063 |
|  | Tryptophan |  | F(1,22)=13.12, p=0.0015 |
|  | Kynurenine |  | F(1,22)=13.33, p=0.0014 |
|  | 5HTP |  | F(1,22)=20.5, p=0.0002 |
|  | 5HT |  | F(1,22)=5.92, p=0.0236 |
|  | 5HIAA |  | F(1,22)=9.8, p=0.0049 |
|  | 5HIAA/5HT |  | F(1,22)=10.78, p=0.0034 |
|  | Tyrosine |  | F(1,22)=6.93, p=0.0152 |
|  | DOPAC |  | F(1,22)=3.87, p=0.0620 |
|  | GSH |  | F(1,22)=6.15, p=0.0213 |
|  | H-Cysteine |  | F(1,22)=11.68, p=0.0025 |
|  | Cysteine |  | F(1,22)=3.82, p=0.0634 |
|  |  |  |  |
| **Male STR FeS** |  |  |  |
|  | Glutamine |  | F(1,21)=5.35, p=0.0309 |
|  | Glutamate |  | F(1,21)=10.31, p=0.0042 |
|  | GABA |  | F(1,21)=9.96, p=0.0048 |
|  | Tryptophan |  | F(1,21)=13.23, p=0.0015 |
|  | Kynurenine |  | F(1,21)=33.1, p<0.0001 |
|  | 5HTP |  | F(1,21)=20.95, p=0.0002 |
|  | 5HT |  | F(1,21)=36.49, p<0.0001 |
|  | 5HIAA |  | F(1,21)=42.15, p<0.0001 |
|  | 5HIAA/5HT |  | F(1,21)=25.94, p<0.0001 |
|  | Tyrosine |  | F(1,21)=15.64, p=0.0007 |
|  | HVA |  | F(1,21)=17.80, p=0.0004 |
|  | DOPAC |  | F(1,21)=8.94, p=0.0070 |
|  | HVA/DA |  | F(1,21)=31.99, p<0.0001 |
|  | DOPAC/DA |  | F(1,21)=4.69, p=0.0419 |
|  | GSH |  | F(1,21)=10.06, p=0.0046 |
|  | H-Cysteine |  | F(1,21)=11.06, p=0.0032 |
|  | Cysteine |  | F(1,21)=13.17, p=0.0016 |
|  | Methionine |  | F(1,21)=4.67, p=0.0423 |
|  |  |  |  |
| **Males FC Fe** |  |  |  |
|  | Glutamine |  | F(1,22)=8.83, p=0.0071 |
|  | Glutamate |  | F(1,22)=12.65, p=0.0018 |
|  | GABA |  | F(1,22)=7.12, p=0.0140 |
|  | Glu/GABA |  | F(1,22)=4.18, p=0.0531 |
|  | Tryptophan |  | F(1,22)=11.48, p=0.0026 |
|  | Kynurenine |  | F(1,22)=12.63, p=0.0019 |
|  | 5HT |  | F(1,22)=12.32, p=0.0020 |
|  | 5HIAA |  | F(1,22)=3.8212.26, p=0.0020 |
|  | 5HIAA/5HT |  | F(1,22)=3.58, p=0.0716 |
|  | Tyrosine |  | F(1,22)=3.78, p=0.0649 |
|  | GSH |  | F(1,22)=8.15, p=0.0092 |
|  |  |  |  |
| **Males STR Fe** |  |  |  |
|  | Tryptophan |  | F(1,22)=24.00, p<0.0001 |
|  | Kynurenine |  | F(1,22)=11.58, p=0.0026 |
|  | H-Cysteine |  | F(1,22)=5.42, p=0.0287 |
